# Diagnosis and Management of Multiple Sclerosis in Children

**Published:** 2016

**Authors:** Mohammad Reza NAJAFI, Mohammad Amin NAJAFI, Zahra NASR

**Affiliations:** 1 Department of Neurology, Isfahan University of Medical Sciences, Isfahan, Iran; 2 Medical student, Isfahan University of Medical Sciences, Faculty of Medicine, Isfahan, Iran; 3 Isfahan Neurosciences Research Centre (INRC), Isfahan University of Medical Sciences, Isfahan, Iran

**Keywords:** Disease-modifying therapies, Pediatric MS, Multiple sclerosis, Treatment

## Abstract

Growing evidence indicates the safety and well toleration of treatment by Disease-modifying in children suffering multiple sclerosis (MS). The treatment is not straight forward in a great number of patients, thus patients with pediatric MS must be managed by experienced specialized centers. Common treatments of multiple sclerosis for adults are first-line therapies. These therapies (firstline) are safe for children. Failure in treatment that leads to therapy alteration is almost prevalent in pediatric MS. Toleration against current second-line therapies has been shown in multiple sclerosis children. Oral agents have not been assessed in children MS patients. Although clinical trials in children are insufficient, immunomodulating managed children, experience a side effect similar to the adult MS patients. However, further prospective clinical studies, with large sample size and long follow-up are needed to distinguish the benefits and probable side effects of pediatric MS therapies.

## Introduction

Multiple sclerosis (MS) is the immune-mediated demyelinated disease of the human-beings that mostly entangle young adults. Nevertheless, children MS estimated to be 10% of all cases. Clinical and laboratory findings have shown differences in the disease characteristics and response to the treatment among children and adults (1, 2). In general, pediatric MS less than 10 yr old occurred rarely and constitutes approximately 20% of the reported pediatric cases in large series (3). There are increasing evidences about epidemiology, pathophysiology, diagnosis, and management of MS in children. Previous studies mainly investigated the role of genetic and environmental factors. Subtypes of Human Leukocyte Antigen (HLA) as well as exposure to viruses are the major potential candidates (4). Pediatric MS is more benign than adult MS. However, because of early age of onset, patients with pediatric MS achieve a noticeable degree of disability earlier than adult MS patients. Treatment of children with MS is mainly based on adult’s treatment (5). Retrospective data have indicated the efficacy of these treatments. An early initiation of treatment with disease modifying drugs in adults with relapsing remitting MS (RRMS), cause persistence slower development of disease (6). Yet, no randomized controlled trials of disease modifying therapies in children have been carried out (2, 7).Management of pediatric MS is a controversy topic that recently attracted the attention. In this article we will review current management of pediatric MS.

**Diagnosis**

Because of the broader spectrum of other childhood disorders with similar symptoms and characteristics, diagnosis of MS in children is more difficult in comparison with adult population (8). In addition, atypical clinical, biological, and MRI presentations cause more difficulties in distinguishing children MS patients (9). MS is historically defined as neurologic symptoms disseminated in space (DIS) and time (DIT). The diagnostic criteria for MS in adults have been changed during past years (10-12). The current McDonald diagnostic criteria may be accepted to children, if the beginning presentation is not identified by encephalopathy. Recently, formulated diagnostic criteria for pediatric MS may help to develop diagnostic precision for the clinicians. The International Pediatric MS Study Group (IPMSSG) published operational definitions for acquired demyelinating diseases of the CNS in children, in 2007 (13). According to definitions of the IPMSSG diagnosis of pediatric MS may be after two separated clinical episodes of demyelination in central nervous system (CNS) by at least 30 days (14). Table 1 shows the last diagnostic criteria mentioned for multiple sclerosis in children (The 2010 McDonald Criteria for Diagnosis of MS).

**Treatment**

**First line disease modifying therapy**

**Disease modifying therapy (DMT)**

First-line disease modifying therapies (interferon beta and glatiramer acetate) were initially approved for use in adults 15–20 yr ago and have since been safely used in children. Pediatric MS has been related with noticeable cognitive impairment (15) as well as lesions on MRI (8). In most cases, DMT can decrease the relapse rate and progression of the MS (16). Decreased probability of presentation with a later clinical relapse was the result of early treatment with DMT in pediatric MS (17). However, usage of DMT in MS patients whose symptoms of their first attack (clinically isolated syndrome) resolvedcompletely with a low lesion load(<6 T2-bright lesions,<2 gadolinium enhancing lesions) is not recommended (18). DMT therapy is recommended if a second attack occurred within every 6 months follow up during two yr or in condition of active disease signs. four firstline (glatiramer acetate (GA), intramuscular (IM) and subcutaneous (SC) interferon β-1a, and SC interferon β-1b), and two second-line therapies (mitoxantrone and natalizumab) are disease modifying therapies accepted for the management of relapsing–remitting MS (RRMS) for adult patients. Priority of these treatments has not been evaluated by randomized controlled trials. Additionally from country to country drug choice is different (6) (Table 2).

**Interferon beta (lNFB)**

Previous studies suggested that INFB is able to effect on MS through inhibition of proinflammatory cytokines, induction of anti-inflammatory mediators, reduction of cellular migration and inhibition of autoreactive T cells (19). Safety and toleration of subcutaneous and intramuscular INFB-1a and INFB-1b in pediatrics MS patients has been shown in retrospective and open label studies (20, 21). No data are available about the effect of INFB on MRI lesions and its usefulness on slowing down the process of disease in children. Side effects of INFB have been reported as: 1. Flu-like symptoms which reported in a great number of MS children (35%- 65%). 2. Leucopenia (8%–27%), 3. thrombopenia (16%), 4. anemia (12%) and 5. transient elevation in transaminases (10%–62%) (21, 22). Liver function tests (LFTs) in younger children taking interferon obviously showed abnormality (20). IPMSSG has recommended that the adjusted dosage of INFB should be used in pediatric MS patients, especially in those under 10 years old. The starting dose should be 25 to 50% of the dosage administrated to adult with a gradually increasing every 2-4 weeks to reach optimum tolerable dose. In children under 10 years old, the treatment is initiated with 10% of the dosage which started in adult and gradually reaches to the maximum tolerable dose (Table 2).

**Glatiramer acetate (GA)**

Glatiramer acetate is a heterogenous mixture ofsynthetic polypeptides consisting of four amino acids (L-alanine, L-glutamic acid, L-lysine, L-tyrosine) found in myelin basic protein. Role of GA is to act as human myelin basic protein, activate myelin specific response of suppressor T lymphocytes and inhibit specific T lymphocytes. The therapy including daily subcutaneous administration of 20 mg GA and according to IPMSSGthere is no need to increase the dosage (23). The safety and well toleration of drug in pediatric MS patients is reported (14, 24). Three retrospective studies have been published assessing the efficacy of GA in pediatrics (16, 24, 25) (Table 2).

**Side effects**

**IFN**

IFN Transient flu-like symptoms experienced by lots of patients during the first trimester of INFB therapy. Headache, transient elevation of the liver enzymes, leucopenia, anemia, thrombocytopenia, and thyroid dysfunction require monitoring during the process of treatment with IFN. These side effects are diminished usually by reduction of drug dosage. Exacerbation of depression symptoms by IFN should be mentioned in depressed patients and thus it is better to avoid IFN usage in these patients. Before administration of INFB, patients who suffered flu-like symptoms can be treated with ibuprofen 10 mg/kg (6, 22, 25, 26) (Table 2).

**GA**

Most common side effects is pain and indurations at the site of injection and transient systemic reactions (23). Transient skin retraction at the injection site as well as chest pain and flushing are complications happening immediately after the injection (21, 24)(Table 2).

**Treatment in condition of exacerbation**

**Methyl Prednisolone pulse**

Methyl prednisolon is an important drug in treatment of MS, especially in the acute phase of relapse. Decreasing the inflammatory process done through different pathways: dampening the inflammatory cytokine cascade, inhibiting the activation of T cells, decreasing the extravasations of immune cells into the central nervous system, facilitating the apoptosis of activated immune cells, and indirectly decreasing thecytotoxic effects of nitric oxide and tumor necrosis factor alpha (27)(Table 2). Exacerbations in children have not been carried out yet, as far as we know. Thus, treatment for acute MS in the pediatric is extremely based on adult treatment. A study of US physicians recommend that many follow the management with the regimen of IV methylprednisolone 20–30 mg/kg/d (up to 1 gm) for 3–5 days for acute MS exacerbations. Notably, not all children who experience acute, receive treatment. Supportive care may be recommended by some physicians if clinical manifestations are mild and do not lead to disability (28) (Table 2).

**Intra Venous Immuno Globulin (IVIG)**

Some case reports which try to manage with IVIG therapy in difficult managing cases of acute demyelinating in children patients with MS, suggest the probable improvement followed by mentioned therapy. However, patients were just those children with both optic neuritis and ADEM (29, 30) (Table 2).

**Plasmapheresie**

Plasma exchange (PE) is an efficient therapy for severe relapses of acute inflammatory CNS demyelinating diseases in adults. Therapeutic plasma exchange (TPE) has been useful in the treatment of several pediatric diseases. However, the safety and efficacy of this management in pediatric patients need to be more developed (31)(Table 2).

**Therapy evaluation**

The patients follow up should be done within 1, 3 and 6 month after therapy and then every 6 month. Efficacy of treatment was evaluated by following the patients in mentioned time and performing the neurological examination. In addition, before starting the uptake of DMT, MRI scan must be performed. MRI scan should be repeated yearly in patients with stable condition (23). If serious side effects are appearing or when side effects are not tolerated by patients and leading to reduced compliance, alteration in treatment is necessary. Furthermore, when the therapy is not as effective as expectation, drug choice should be changed. Yet, nostudies or suggestion for pediatric MS are available to guide the decision if therapy should be changed or not (6). In patients who are not able to tolerate the side effects of DMT, the treatment should be changed from INFB to GA or vice versa. No overlap or time delay between discontinuing the first drug and initiation of second one is acceptable. Dosage of INFB should be gradually increased as it introduced first as described above. Supporting the suboptimal response to DMT is based on the above mentioned suggestion and needs at least 6 month time after the starting the treatment. However, individual decision for each patients must be taken as there is not follow a strict algorithm (32).

**Failure in treatment: Second-line therapies**

Therapy failure is a concern in the pediatric MS individuals. Failure in treatment is defined differently with a high controversy among physicians treating adult-onset MS.Six US Pediatric MS Centers of Excellence followed the 258 children with MS, 123 (48%) children failed in their first line of treatment. Noncompliance and intolerable side effects were observed in a notable percentage of these patients. Thirty seven (16%) of patients switched their therapies as a result of noncompliance or side effects, 72 (28%) because of progression of disease, and 4% (12) due to discontinuation of treatment after the first agent (33). Yet, there is not any approved algorithm for the treatment of partial responsiveness, natalizamab, cyclophosphamide, mitoxantrone, and rituximab are the immunomodulatory and cytotoxic agents reported in pediatric MS patients resistant to therapy (34).

**Table 1:** Last Diagnostic Criteria Mentioned for Multiple Sclerosis in Children (The 2010 McDonald Criteria for Diagnosis of MS)

**Clinical presentation**	**Additional data needed for MS diagnosis**
≥2 Attacks^a^; objective clinical evidence of >2 lesions or objective clinical evidence of 1 lesion with reasonable historical evidence of a prior attack^b^	None^c^
≥2 Atracks^a^; objective clinical evidence of one lesion	Dissemination in space, demonstrated by: ≥1 T2 lesion in at least 2 of 4 MS-typical regions of the CNS(periventricular, juxtacortical, infratentorial, or spinal cord)^d^: or Await a further clinical attack ^a^ implicating a different CNS site
≥1 Attack ^a^: objective clinical evidence of 2 lesions	Dissemination in time, demonstrated by: Simultaneous presence of asymptomatic gadolinium-enhancing and non enhancing lesions at any time: or A new T2 and/or gadolinium-enhancing lesion(s) on follow-up MRI, irrespective of its timing with reference to a baseline scan; or Await a second clinical attack^a^
≥1 Attack^a^: objective clinical evidence of 1 lesion (clinically isolated syndrome)	Dissemination in space and time, demonstrated by: For DIS: 1 T2 lesion in at least 2 of 4 MS-typical regions of the CNS (periventricular, Juxtacortical, infratentorial, or spinal cord)^d^; or Await a second clinical attack^a^ implicating a different CNS site: and For DIT: Simultaneous presence of asymptomatic gadolinium-enhancing and nonenhancing lesions at any time; or A new T2 and/or gadolinium-enhancing lesion(s) on follow-up MRI irrespective of its timing with reference to a baseline scan; or Await a second clinical attack^a^
Insidious neurological progression suggestive of MS (PPMS)	One year of disease progression (retrospectively or prospectively determined) plus 2 of 3 of the following criteriad: 1. Evidence for DIS in the brain based on 1 T2 lesions in the MS characteristic (periventricular, juxtacortical, or infratentorial) region 2. Evidence for DIS in the spinal cord based on 2 T2 lesions in the cord 3. Positive CSF (isoelectric focusing evidence of oligoclonal bands and/ or elevated IgG index)

If the criteria are fulfilled and there is no better explanation for the clinical presentation, the diagnosis is “MS”; if suspicious, but the criteria are not completely met, the diagnosis is “possible MS”; if another diagnosis arises during the evaluation that better explains that clinical presentation, then the diagnosis is “not MS.”

a : An attack (relapse; exacerbation) is defined as patient reported or objectively observed events typical of an acute inflammatory demyelinating event in the CNS, current or historical, with duration of at least 24 h, in the absence of fever or infection. It should be documented by contemporaneous neurological examination, but some historical events with symptoms and evolution characteristic for MS, but for which no objective neurological findings are documented, can provide reasonable evidence of a prior demyelinating event. Reports of paroxysmal symptoms (historical or current) should, however, consist of multiple episodes occurring over not less than 24 hs. Before a definite diagnosis of MS can be made, at least 1 attack must be corroborated by findings on neurological examination, visual evoked potential response in patients reporting prior visual disturbance, or MRI consistent with demyelination in the area of the CNS implicated in the historical report ofneurological symptoms.

b : Clinical diagnosis based on objective clinical findings for 2 attacks is most secure. Reasonable historical evidence for 1 past attack in the absence of documented objective neurological findings can include historical events with symptoms and evolution characteristics for a prior inflammatory demyelinating event; at least 1 attack, however, must be supported by objective findings.

c : No additional tests are required. However, it is desirable that any diagnosis of MS be made with accessto imaging based on these criteria. If imaging or other tests (for instance, analysis of CSF) are undertaken and are negative, extreme caution needs to be taken before making a diagnosis of MS, and alternative diagnoses must be considered. There must be no better explanation for the clinical presentation, and objective evidence must be present to support a diagnosis of MS.

d : Gadolinium-enhancing lesions are not required; symptomatic lesions are excluded from consideration in subjects with brain stem or spinal cord syndromes.

**Table 2:** Brief Review of Drugs Used in Treatment of Multiple Sclerosis

**Drug**	**Administration**	**Dosage**	**Side effect (most frequents)**	**Mechanism**
Interferon beta-la (Avonex)†	Intramuscular (IM)	30 mcg Once weekly	Transient flu-like symptoms	1.Inhibition of proinflammatory cytokines, 2.Induction of anti-inflammatory mediators, 3.Reduction of cellular migration and 4.inhibition of autoreactive T cells
Interferon beta-1a (Rebif): †	Subcutaneous (SC)	22 or 44mcg three times a week		
Interferon beta-1b (betaferon)	Subcutaneous (SC)	8 million international units/ every other day		
Glatiramer acetate †	Subcutaneous (sc)	20 mg Daily	Pain and indurations at the site of injection	1.Act as human myelin basic protein, 2.activate myelin specific response of suppressor T lymphocytes 4. Inhibit specific T lymphocytes.
Methyl Prednisolone pulse	Intra venous	20–30 mg/kg/d for 3–5 days for acute MS exacerbations	Infection, Chronic Trouble Sleeping, Conditions of Excess Stomach Acid Secretion, Nervous	1.Dampening the inflammatory cytokine cascade 2. Inhibiting the activation of T cells 3.Decreasing the extravasation of immune cells into the central nervous system 4.Facilitating the apoptosis of activated immune cells 5. Indirectly decreasing the cytotoxic effects of nitric oxide and tumor necrosis factor alpha.
Natalizumab (Tysabri)	Intravenous	3-5mg/kg monthly	Progressive multifocal leukoencephalopathy (PML)	1.Monoclonal antibody that targets α4β1-integrin 2. Effectively blocks T- and B-cell migration across the bloodbrain Barrier
Cyclophosphamide (Cytoxan)	Intravenous/orally	600–1000 mg/m^2^ per dose	Vomiting, transient alopecia, osteoporosis, and amenorrhea	1.Binds to DNA and interferes with mitosis and cell replication 2. Decrease the secretion of the pro-inflammatory T helper (Th) 1 cytokine interferon-γ (IFN_γ_) and interleukin (IL)-12 3.Increase the secretion of the anti-inflammatory Th2 cytokines IL-4 and IL-10 in cerebrospinal fluid (CSF) and peripheral blood
Rituximab¥ (Rituxan)	Intravenous	Maximum dose 1000 mg per infusion, total dose per cycle range, 416- 1168 mg/m2 twice 2 weeks apart §	Progressive multifocal leukoencephalopathy (PML)	1.Antibody that primarily targets the CD20 receptor on activated B cells
Daclizumab¥	Intravenous	1.0 mg/kg	Elevated liver function tests, infections, psoriasis and oral ulcers	1. Inhibiting T-cell replication 2.Making more IL-2 available to the low-affinity CD25 receptor present on NK cells, which induces a regulatory NK cell population
Fingolimod¥ (Gilenya)	Orally	0.5 mg once daily	Headache, influenza, diarrhea, back pain, liver transaminase elevations, and cough	1.Targets the sphingosine-1-phosphate receptor that is necessary for lymphocyte egress from lymph nodes 2.Target Th17 central memory cells

† The recommended doses of these drugs are similar in both adults and adolescents heavier than 50 kg; but, for children less than 10 yr of age, the dose is calculated based on the child weight in kilograms divided by 50kg and multiplying the results by adult dose (16, 21).

¥ The mentioned dose is for adult patients and there is no study to approve these dosage for pediatric multiple sclerosis children.

§ adapted from adult and pediatric rheumatology studies (67).

**Escalation therapy**

Cyclophosphamide, mycophenolatemofetil, daclizumab, mitoxantrone, rituximab and natalizumab have been used in pediatric MS patients (35-37). However, their prescription in children has not been suggested yet.

**Natalizumab**

Natalizumab is a humanized monoclonal antibody that targets the a4 subunit of a4b1 and 4b7 integrins, molecules involved in the transmigration of immunecells into the CNS (38). Natalizumab has been accepted for the management of relapsing forms of MS (RRMS) (39). Adult MS patients who require an escalation therapy are treated with natalizumab with a dosage of 3-5 mg/kg as an infusion every 4 wk. In only one pediatric study performed in Italy, natalizumab (300 mg, every 28 days) was used in 19 children with severe active MS. After 15-month follow up relapses and MRI lesions suppressed in all patients and no serious side effects were reported (40). In Portugal, 383 cases were enrolled in natalizumab therapy and treatment with natalizumab was more effective in patients with less disability and without prior disease modifying therapy (41). However, natalizumab is not approved for patients less than 18 yr old and in some cases leads to progressive multifocal leukoencephalopathy (PML). Thus, natalizumab therapy must be restricted to specialized centers (6, 35, 37, 40, 42)(Table 2).

**Cyclophosphamide**

Cyclophophamide has not been approved for the treatment of MS. But, prescription of pulse cyclophosphamide decrease disease activity in adult MS patients (43, 44). In a retrospective study, 17 children aged 9 to 18 yr were treated with cyclophosphamide at either pulse or induction therapy most of the cases indicated improvement of relapse frequency and EDSS 1 yr after the initiation of cyclophosphamide therapy (37)(Table 2).

**Rituximab**

Rituximab is not accepted for the treatment of MS, but positive effects has been indicated in adult RRMS that showed noticeable decrease of brain lesions and clinical relapses. A recent study assesses 8 pediatric neuromyelitisoptica and 3 MS patients and reported that the use of rituximab in our pediatric neuromyelitisoptica and multiple sclerosis cohort was overall safe and effective (45-48)(Table 2).

**Daclizumab**

Intravenous daclizumab, has been used off-label in adult MS patients (49-52) and another subcutaneous form is being tested in clinical trials in adult MS. Recently, a study assessed the efficacy intravenousdaclizumab in 7 pediatric MS patients treated largely in combination with beta interferons. This study indicated that treatment with daclizumab was in association with reductions in ARR, number of contrast enhancing lesions, and reduction or stabilization of EDSS in each patient. However, 4 patients had relapses and new contrast enhancing lesions during daclizumab treatment (53)(Table 2).

**Side effect**

**Natalizumab:** Hypersensitivity reactions and development of antibody to the drug. The most important side effect is a 1:1000 risk of progressive multifocal leukoencephalopathy (PML) observed in adult patients (54-56).

**Cyclophosphamide:** Side effects of cyclophosphamide include vomiting, transient alopecia, osteoporosis, and amenorrhea. Development of bladder carcinoma has been seen in one patients that was successfully treated (57, 58).

**Rituximab:** Probable side effect of rituximab is development of PML and other severe infection (50- 52)

**Daclizumab:** Elevated liver function tests, infections, psoriasis and oral ulcers are adverse effects related to intravenous daclizumab treatment (50-53, 59, 60) (Table 2).

**Oral agents**

Fingolimod, and cladribine for adult MS patients, influence the lymphocytes by various mechanisms. Yet, no study about usage of these two oral agents in pediatric MS patients has been published. However, negative effects founded in association with these agents in adult MS patients, consisting of cancer and lethal herpetic infections. Thus, adopting these therapies in pediatric population requires serious attention (61-63).

**Fingolimod**

Fingolimod is an orally administered small molecule that targets the sphingosine-1-phosphate receptor that is necessary for lymphocyte egress from lymph nodes. Fingolimod was approved for adult patients with MS. No information currently exists about fingolimod with regard to safety, tolerability and dosage in children (62,64-66)(Table 2).

**In conclusion,** Children appear to accrue locomotor disability more slowly, but they can have significant cognitive deficits, even early on in the course of the disease. There have been no randomized-controlled studies of disease-modifying therapies. However, firstline therapies, beta interferon, and glatiramer acetate are extensively used off-label. Therapies in pediatric MS are being developed and may be implemented in the next few years.
